# A Qualitative Investigation on Patient Empowerment in Prostate Cancer

**DOI:** 10.3389/fpsyg.2017.01215

**Published:** 2017-07-26

**Authors:** Chiara Renzi, Chiara Fioretti, Serena Oliveri, Ketti Mazzocco, Dario Zerini, Ombretta Alessandro, Damaris P. Rojas, Barbara A. Jereczek-Fossa, Gabriella Pravettoni

**Affiliations:** ^1^Applied Research Division for Cognitive and Psychological Science, European Institute of Oncology Milan, Italy; ^2^Interdisciplinary Research and Intervention on Decision, Department of Oncology and Hemato-Oncology, University of Milan Milan, Italy; ^3^Division of Radiation Oncology, European Institute of Oncology Milan, Italy; ^4^Department of Oncology and Hemato-Oncology, University of Milan Milan, Italy

**Keywords:** prostate cancer, radiation therapy, decision-making, patient empowerment, eHealth, personalized medicine, unmet needs

## Abstract

**Purpose:** Men with prostate cancer often describe low levels of empowerment. eHealth interventions may represent useful tools to deliver care and education and to meet patients' needs within an empowerment framework. In order to design a platform for cancer patients' empowerment within the H2020 iManageCancer project, the perspective of the target population for the platform was assessed. The present study aims to assess the qualitative experience of prostate cancer patients during treatment in order to provide insights for clinical practice with a particular focus on the design of a web platform to promote cancer patients' empowerment.

**Methods:** Ten patients undergoing radiation therapy treatment took part in a semi-structured interview to explore different aspects of patient empowerment. Four main thematic areas were addressed: patient-healthcare providers' communication, decision-making, needs, and resources. A qualitative approach using thematic analysis was followed.

**Results:** Half of the patients reported little to no possibility to share information and questions with healthcare providers. With regards to decision-making, the role of healthcare providers was perceived as directive/informative, but half of the patients perceived to assume an active role in at least one interaction. Difficulties and needs included the choice of the specialist or of the structure after diagnosis, clinicians' support in self-management, surgical consequences, and side effects, preparation for radiation therapy. Resources included family and social support both from a practical and from an emotional perspective, coping style, and work schedule management.

**Conclusions:** These results suggest that relations with healthcare providers should be supported, especially immediately after diagnosis and after surgery. Support to self-management after surgery and at the beginning of radiation therapy treatment also constitutes a priority. The adoption of a personalized approach from the beginning of prostate cancer care flow may promote patient empowerment, overcoming the aforementioned needs and mobilizing resources. The social network represents an important resource that could be integrated in interventions. These considerations will be taken into account in the design of a cancer self-management platform aiming to increase patients' empowerment.

## Introduction

Web-based and eHealth interventions revealed to be promising tools in order to deliver care and education and to meet patients' needs within an empowerment framework (Ventura et al., 2013; Wolpin et al., 2014; Lubberding et al., 2015; Wheelock et al., 2015). Further studies need to be conducted in order to test the efficacy of personalized eHealth platforms in promoting patient empowerment (Violette et al., 2015). These tools are often designed by clinicians or app developers, hence they are proposed to patients for use. Nevertheless, recent works pointed out the importance to start from needs assessment of the target population prior to the design and the delivery of the intervention (Friedman et al., 2011; Leykin et al., 2012; Ventura et al., 2013).

The European H2020 project iManageCancer (grant agreement no. 643529) aims to develop and test the efficacy of a web-based eHealth platform for mobile devices composed by apps and services to improve self-management abilities and to promote cancer patients' empowerment. In accordance with the aforementioned indications, the works for the project started with the assessment of different aspects of patient empowerment amongst individuals with prostate cancer, the target population for the pilot study with the platform.

Prostate cancer is the most prevalent male cancer in Europe, and the second most prevalent male cancer worldwide (Ferlay et al., 2013). This cancer is often diagnosed at an early stage (59% at stage I or II), with almost no change in survival rates compared to the general population (112% for stage I and 99% for stage II). Even considering patients diagnosed at all stages of the disease, 84% of men with prostate cancer survive at least 10 years after diagnosis, thus pointing to survivorship and quality of life as important issues for this malignancy (Davies and Batehup, 2010; Bourke et al., 2015; Gilbert et al., 2015).

Depending on the stage, on the characteristics of the disease, and on the general health status of the patient, different options are available for localized prostate cancer such as surgery, radiation therapy, hormonal therapy, active surveillance, and watchful waiting (Dahm et al., 2008; Horwich et al., 2013). These treatments have diverse short and long term side effects, which demand careful consideration for their possible impact on life-style, sentimental and sexual life, body image, and psychological well-being (Pravettoni and Gorini, 2011; Bourke et al., 2015). Men with prostate cancer experience low levels of empowerment and self-management abilities (Davison and Degner, 1997; Harrison et al., 2009; Cockle-Hearne and Faithfull, 2010; Watson et al., 2016). For instance, they often remain passive in front of the treatment choice (Davison et al., 1995; Davison and Degner, 1997), and report information and communication as unmet needs (Davison et al., 1995; Lintz et al., 2003; Harrison et al., 2009; Watson et al., 2016).

Starting from these premises, the present study aims to explore issues related to communication, involvement in the treatment decision-making process, unmet needs and resources of prostate cancer patients in order to design a cancer platform that may support patient empowerment. Furthermore, it can provide preliminary indications to clinicians regarding the patients' view on treatments, their expectations and their difficulties.

The majority of studies assessing supportive care needs in men with prostate cancer used surveys and validated questionnaires (e.g., Cockle-Hearne et al., 2012), while qualitative techniques are used less frequently (see Paterson et al., 2015 for a review). The present assessment investigated the qualitative experience of living with and undergoing radiation therapy for prostate cancer, in order to unveil aspects that otherwise may be overlooked in quantitative analyses.

## Materials and methods

### Participants

Participants were enrolled at a comprehensive cancer center in northern Italy within a period of 2 months in 2016. Inclusion criteria were: (i) age between 40 and 75 years old; (ii) diagnosis of non-metastatic prostate cancer; (iii) absence of major psychiatric disorders; (iv) undergoing external beam radiotherapy for prostate cancer; (v) written informed consent for the study.

The choice of recruiting patients undergoing radiotherapy was related to the consideration that in this phase patients performed already a sufficient number of consults with different clinicians, and are not too close to the moment of diagnosis yet still in treatment.

This study was carried out in accordance with the recommendations of Good Clinical Practice guidelines (International Conference on Harmonisation, 2016) with written informed consent from all subjects. All subjects gave written informed consent in accordance with the Declaration of Helsinki. The protocol was approved by the Ethical Committee of the European Institute of Oncology.

Patients fitting inclusion criteria were approached by their treating physicians, and proposed to take part in the study after explaining the study aims and procedures.

An overall number of 21 consecutive prostate cancer patients responding to inclusion criteria were contacted over a one-month period. Eleven patients refused due to time constraints (either for them or for the person who accompanied them to therapy) related to family or work organization or because they were not interested in the study. Ten patients gave their consent and underwent the semi-structured interview (see Tables 1, 2 for characteristics).

**Table 1:** Socio-demographic and clinical characteristics by patient.

**No**.	**Residence**	**Marital status**	**Education**	**Comorbidities**	**Prostatectomy**
1	Lombardy	Married	University	Ischemic cardiopathy (PTCA), Hemorrhoids	Yes
2	Milan Area	Widowed	High School	Pulmonary arterial hypertension	Yes
3	Other region	Married	University	Ascending aortic ectasia	No
4	Lombardy	Married	Middle school	Carotid artery stenosis, chronic gastritis, right hemicolectomy (right colon cancer)	No
5	Other region	Married	High school	Nephrolithiasis	Yes
6	Milan Area	Married	Middle School	Arterial hypertension	No
7	Other region	Married	Middle School (+ 2 years)	Mitral valve prolapse	Yes
8	Milan Area	Married	University	Arterial hypertension	Yes
9	Other region	Separated	University	None	Yes
10	Lombardy	Married	Middle School	Pulmonary arterial hypertension, multiple myeloma (stem cell transplant), chronic kidney disease (solitary kidney)	Yes

*PTCA, Percutaneous transluminal coronary angioplasty*.

**Table 2:** Summary of clinical characteristics.

**Characteristics**	**All patients, *N* = 10**
**AGE IN YEARS**	
Median (range)	67.5 (56–71)
**STAGING**	
T2a	1
T2c	3
T3a	2
T3b	3
T4	1
**INITIAL GLEASON SCORE**	
Median (range)	7 (6–9)
**ANTIPLATELET THERAPY**	**2**
**TARGET**	
Whole prostate	3
Tumor bed	7
**TYPE OF SURGERY**	
RPNNS+PLND	4
RPNS+PLND	2
RPNS	1
**SURGERY PERFORMED**	
On site	4
Other centers	3
**CONCOMITANT ADT (LHRH AGONISTS)**	**4**
**RADIOTHERAPY INTENT**	
Radical	3
Adjuvant	3
Salvage	4

*RPNNS, radical prostatectomy not nerve sparing; RPNS, radical prostatectomy nerve sparing; PLND, pelvic lymph node dissection; ADT, androgen deprivation therapy; LHRH, Luteinizing hormone-releasing hormone*.

Four patients underwent surgical treatment in the same hospital where the study was conducted. The other three underwent surgical procedures elsewhere. Three patients did not have surgery.

### Data collection

The methodology of the study was divided into three phases.

In the first explorative phase, as a result of non-formal contacts with the Head and the members of the oncological division for prostate cancer care, the researchers examined the ward procedures and interpersonal dynamics experienced by patients from the professionals' point of view. Some of the authors of this study (CR, CF, SO, and KM with the support of DZ and BJ) investigated the therapeutic pathways of prostate cancer patients at the institution where the study was conducted, by conducting individual meetings with the healthcare professionals of the urology and radiation therapy wards. Each professional was asked to narrate the therapeutic pathway of the patients and for each step of the therapeutic pathway to describe the staff involved, possible decisions, timings, and procedures. Meetings were repeated until saturation (no new information reported). The psycho-oncologists were also invited to the weekly meeting of the multidisciplinary equipe for prostate cancer patients and collected professionals' point of view on patients' needs.

Starting from the information gathered during this phase, a meeting of all the psycho-onchologists (CR, SO, CF, KM, GP) was then set to discuss the questions for the interview. The researchers designed the interview structure considering four thematic areas: “Patient-physician quality of communication,” “Quality of decision-making,” “Needs,” and “Resources.” The areas encompassed the following contents:

1. Patient-physician quality of communication: patients' perception of their relationship and communication with the clinical staff. Questions as “Do you think that the information you received from your treating physician corresponded to your knowledge needs? Can you explain why?” or “How would you evaluate the information exchange with the healthcare professionals?” were formulated.
2. Quality of decision-making: patient's perception of his/her role in decision-making for therapies, along with the perception of physician position. Questions as “Were there any moments during the therapeutic pathway when you had to take decisions?” If so, “did you feel able to do it? Did you want to?” were formulated.
3. Needs: practical or psycho-emotional needs experienced throughout the care flow. Questions as “Do you think there were/are important needs which are currently not taken into consideration?” or “Did you experience difficulties in the relational area or in managing your disease? When? Of which type?” were formulated.
4. Resources: individual or systemic resources employed to cope with the disease and the treatments. Questions as “Which resources did you have to use throughout the different steps” or “With respect to the difficulties you described, what was done to overcome them?” were formulated.

In the second phase, data collection was initiated. Semi-structured interviews were conducted with each patient by a psycho-oncologist, and lasted 45–75 min. All patients were undergoing radiation therapy treatment at the time of the study. The interviews were conducted while patients were waiting for therapy or immediately after receiving therapy, in a room made available by the staff and suitable for listening, without preset time limits.

Patients' answers were literally transcribed in order to facilitate data analysis.

### Data analysis

In the third phase of the study, a thematic analysis (Boyatzis, 1998) was performed. In order to code the collected interview, the main theories on models of doctor-patient communication and decision-making process were analyzed (Ong et al., 1995; Fioretti and Smorti, 2014). It is indicated in the literature that there are several models of communication in the relationship between the clinician and the patient (Emanuel and Emanuel, 1992; Elwyn et al., 2012). They differ for the role of the patient in constructing and negotiating the therapeutic pathway of care. In this sense, some models suggest a patient who is the recipient of technical and scientific information on the different therapeutic options, others recognize an active role of the patient which implies a shared communication in which even the patient is the holder of important information needed to construct a personalized therapy (Emanuel and Emanuel, 1992; Ong et al., 1995; Elwyn et al., 2012; Fioretti and Smorti, 2014, 2017). Starting from the abovementioned models and the results of the explorative phase with the professionals involved in the care path, we constructed the following categories of the type of communication and decision-making process.

The preset categories were considered as a guide providing direction for data analysis starting from topics from the research literature (Taylor-Powell and Renner, 2003). In fact, in line with the thematic analysis approach for qualitative data, “thematic analysis is a process for encoding qualitative information. […] The themes may be initially generated inductively from the raw information or generated deductively from theory and prior research” (Boyatzis, 1998, p. vii).

For the “Patient-physician quality of communication” thematic area, two coding categories were constructed namely “Type of communication” and “Patient's role.”

#### Type of communication

– Directive/instructive: The physician gives few information about the therapeutic options and focuses on what to do, without asking opinions or involving the patient. The doctor provides standard information without adapting it to the patient.
– Shared: The doctor shares information adapting the communication to the doubts and needs raised by the patient. The patient is involved in the exchange of contents.

#### Patient's role in communication

– Passive: The patient listens to the options passively undergoing the information flow.
– Active on demand: The patient does not voluntarily intervene in the communication, because e.g., he does not feel able to share specific contents. Nevertheless, he assumes an active role in communication if explicitly invited by the doctor.
– Active: The patient is involved in the exchange of content and feels free to ask questions, express concerns, and raise salient issues.

Similarly, for the “Quality of decision-making” area, “Type of decision” and “Patient's role in decision” categories were constructed.

#### Type of decision

– Directive/informative: The doctor explains treatment options requiring treatment that he deems appropriate.
– Shared: The doctor explains treatment options by adapting the alternatives to the current conditions and needs raised by the patient. The physician involves the patient in decision-making, raising demanding for concerns.

#### Patient's role in decision

– Passive: The patient is not substantially involved in decision-making, because e.g., he does not feel confident or able to decide outside the options given by the doctor or because no decision to be taken by the patient is proposed.
– Shared: Patient and physician share knowledge, information on the clinical situation and context, needs and concerns, building together the decision-making process.
– Active: The patient has access to knowledge or options, expression of needs or concerns but he does not share with the physician the responsibility of the decision.

### Difficulties and needs

Organizational, relational, and psycho-emotional difficulties and needs were reported, along with correspondence between expectation in interactions with the clinical staff and the specific phase of the clinical pathway. The expression of needs and difficulties was grouped into subthemes based on patients' narratives.

### Resources

Organizational, relational, and psycho-emotional resources were reported, along with the specific phase of the clinical pathway. The expression of resources was grouped into subthemes based on patients' narratives.

## Results

The results section is organized based on the thematic areas explored in the semi-structured interview. Within each paragraph, results are presented based on the thematic analysis and on the coding categories (for the thematic areas “Patient-physician quality of communication” and “Quality of decision-making”) or themes extracted from patients' narratives (for the thematic areas “Difficulties and needs” and “Resources”) described in the Data Analysis section.

### Patient-physician quality of communication

Five patients out of 10 reported the possibility to share information and questions with at least one healthcare provider throughout their pathway. These patients also had an active role in communication (see Table 3).

**Table 3:** Overview of communication style, communication role, decision-making style, and decision-making role experienced by patients.

**No**.	**Communication style**	**Communication role**	**Decision-making style**	**Decision-making role**
1	Shared	Active on demand	Shared	Shared
2	Directive/Informative	Passive	Directive/Informative	Passive
3	Directive/Informative	Passive	Directive/Informative	Active
4	Directive/Informative	Passive	Directive/Informative	Passive then Active
5	Directive/Informative then Shared	Passive then Active	Directive/Informative then Shared	Passive then Active
6	Shared	Active	Directive/Informative	Passive
7	Directive/Informative then Shared	Passive then Active	Directive/Informative	Shared
8	Shared	Active	Directive/Informative	Passive
9	Shared	Active	Shared	Active
10	Shared	Active	Shared	Active

For instance, Patient 6 said:

“*I was free to express my own fears explicitly, and I found in the physician a competent acknowledgement. He explained in what consisted the therapies.*”

Patient 8 stated:

“*[about the side effects of therapy] I did not search the internet, I trusted the people that were taking care of me. I did not want to know everything. […] Concerning radical prostatectomy, we discussed the possible side effects of surgery.*”

Patient 9 stated:

“*I feel I had an active role. I could speak, broaden on the topic, examine in depth some aspects with the physicians such as the therapeutic pathway, its length, precautions to take during treatment, possible side effects and when I should call the physician in case of adverse events.*”

Patient 10 responded:

“*The communication was good. I felt that the physician was trustworthy and kind, and I could ask the questions that were important for me and receive answers.*”

Three patients experienced a directive type of communication.

For instance, Patient 2 said:

“*They explained me more or less why I had to do radiation therapy, but no physician ever explained me all the possible options. Maybe I heard different opinions but no one took some time to tell me about these options. Five minutes and then…another patient!*”

Patient 3 reported:

“*This surgeon I met, he told me —*<*This is a prostate that should be removed right away. I know what I have to do!*>*. The doctor I met afterwards was more kind, but he proposed me radiation therapy without explaining me why.*”

Patient 4 said:

“*The doctor was not clear about the specific surgery he wanted to perform; he did not mention the problems that it would cause. He was bland and I felt he was not sincere.*”

All of these patients also had a passive role in communication. For example, Patient 5 reported:

“*I felt I could not express my doubts…maybe it was me not being in the right state.*”

Two patients reported a change in treating physician explicitly linking this to a difference in communication style and his perceived communication role.

Directive/Informative communication style always corresponded to the feeling of not being active or in control of communication. Shared communication was associated with active communication roles.

### Quality of decision-making

The role of healthcare providers was perceived as directive/informative in seven cases (see Table 3). For instance, Patient 2 said:

“*It was Dr. [Name of the Physician] who chose. I said ‘yes, ok’ to the surgery even if I did not understand.*”

Patient 6 answered:

“*I did not have particular decisions to take or alternatives to choose: the clinical pathway was the one prescribed by my physician.*”

Patient 7 said:

“*They explained me how the therapy worked, but then they decided everything. The physician put me in the list, and then he got rid of me in ten minutes. He was brusque.*”

Interestingly, while in communication the style of relation described was congruent with the role in information exchange in nine cases on 10, here different results can be observed. In fact, two patients that experienced directive/informative decision-making reported to assume an active role after a passive one that led to side effects or complications related to treatment. Two other patients described themselves as being active even though the decision-making style was directive. For Patient 3, this was related to the description of a feeling of mistrust (“*I felt they were not being transparent*”) for healthcare providers that led him to take autonomous decisions on treatments. For Patient 7, this was related to the fact he was informed on every aspect of the therapy and thus may have decided even though he did not do so.

The other three patients experienced shared decision-making e.g., Patient 5:

“*Dr. [Name of the Physician] gave us all the information about therapeutic options and suggestions based on his experience. Then my wife and I decided together about the therapy.*”

### Difficulties and needs

**Figure 1:**
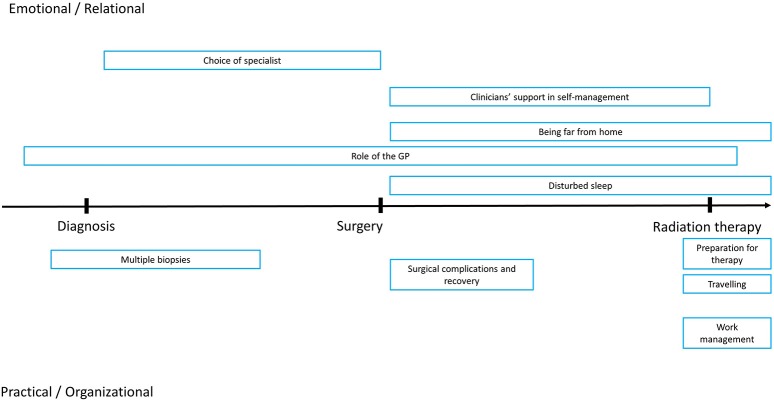
Graphic representation of reported difficulties by temporal dimensions (diagnosis, surgery, radiation therapy) and type (Emotional/relational, Practical/organizational).

To facilitate interpretation of data, difficulties and needs were classified and displayed along two dimensions: one ranging from practical/organizational to emotional/relational, while the other ranging from diagnosis to surgery to radiation therapy (Figure 1).

Perceived role of the general practitioner (3 participants): feeling that the general practitioner underestimated initial symptoms, that he/she did not provide sufficient information to cover information gaps; feeling the absence of a figure that coordinated diagnostic exams, therapeutic plans and follow-ups (Emotional/relational; diagnosis to therapy). Patient 6 said “*The GP underestimated the symptoms, he did not alert me sufficiently*.” Patient 5 reported: “*The GP was not prepared to support my needs*.”

Multiple biopsies (1 participant): choice of an approach that led to performing several biopsies, while the patient did not understand the rationale of this approach and found it painful (Practical/organizational; before and after diagnosis).

Choice of specialist (5 participants): Patients reported relational difficulties with physicians, related to excessive perceived distance or confidence, lack of possibility to express doubts or fears related to the therapeutic approach, lack of sufficient information on some aspects of the therapeutic approaches discussed in the consultation (Emotional/relational; after diagnosis).

For instance, Patient 3 said: “*I experienced relational difficulties with many physicians, they did not behave in a way I felt appropriate to my personality.*” Patient 4 stated: “*The excessive confidence caused a situation where physicians would not tell me everything they had to tell me, it ended up with low transparency.*” Patient 8 said: “*The sexual aspects and side effects were handled in a pretty poor way because we knew each other [referring the physician and the patient]*.” Patient 10 said: “*I would have needed someone to take away wrong fantasies and fill in information gaps.*”

Clinicians' support in self-management (4 participants): fear and worry related to post-surgical procedures or complications, perceived lack of empathy for the worry associated with surgical complications, perceived lack of a reference figure for actual or possible surgical complications, perceived lack of information to determine “normal” or alarming symptoms after the surgical procedure (Emotional/relational; after surgery).

For instance, Patient 7 said: “*I called in the Ward to say that I had pain at the drainage site. They told me it was normal but in a way I felt that they lacked to understand and empathize with my difficulty.*” Patient 9 said: “*I am preoccupied and anxious about the side effects of radiation therapy.*” Patient 2 stated: “*I did not know who I should have called if I felt a burning sensation at the catheter or if I felt it was coming out.*” Patient 5 reported: “*I felt an intense pain and I was frightened.*”

Surgical consequences and surgical complications (6 participants): catheter management, drainage, and urinary incontinence were considered a major issue in the post-surgical phase. Patients reported they felt pain, and could not establish to what extent this was part of the normal post-operative development. In three cases, post-surgical complications were reported. In one case, drug management was cited as a related issue (Practical/organizational; after surgery). For instance, Patient 1 said: “*I had some trouble in managing the catheter and I was incontinent in the first days after surgery*.” Patient 6 reported: “*There were not only diagnostic but also technical mistakes. They placed and removed the catheter after I experienced urine block.*” Patient 4 said: “*I had to repeat surgery twice because the first one was not performed well.*” Patient 2 stated: “*I am worried to become ‘addicted’ to medications. Before it was the painkillers, now the laxatives.*”

Travelling (3 participants): traveling daily or in alternate days to the hospital to receive radiation therapy represents a significant burden, both in terms of expenses as well as in terms of time, especially for those patients who live in other cities or towns. Two patients traveled from their homes, another stayed near the hospital, and traveled back home in the weekends (Practical/organizational; during radiation therapy). For instance, Patient 5 said “*The hospital is far from where I live. Basically, I travel for 3 hours to undergo a therapy that takes 15 minutes*.” Patient 7 stated “*[…] and then I have to reach the hospital on time*.” Patient 9 reported “*The travel to go back home in the weekends was hard at the beginning*.”

Being far from home (1 participant): being far away from home and the support network (Emotional/relational; during radiation therapy).

Work management and security (1 participant): traveling to the hospital represented a burden also in terms of work organization for a patient actively working during radiation therapy (Practical/organizational; during radiation therapy). Patient 5 said: “*I feel that I would complicate the life of my colleagues taking days or hours off for therapy, so I arrange for therapy outside working hours. I am also worried that if I didn't do this I might lose my job, as I don't have a stable contract*”.

Preparation for radiation therapy (4 participants): the necessity to perform therapy with a full bladder and empty rectum was reported to be a significant cause of distress. Time schedules for therapy were described as being too variable. At the same time, patients did not feel sufficiently instructed or efficient in preparation (Practical/organizational; during radiation therapy). Patient 2 said: “*In order to have the bladder full for therapy, it is pivotal to know about the timings, so that I know when I should start drinking. […] I would appreciate if I could say at my arrival that I feel my rectum full so that I can ask for the enema right away at my arrival instead of waiting until the time of my therapy*.” Patient 3 added: “*Therapy with a full bladder cannot be postponed for 1 hour. It should be calibrated better and patients should be instructed about this*.”

Disturbed sleep (1 participant): side effects of surgery resulted in the necessity to urinate often during the night. For one patient this was considered a source of distress (Emotional; after surgery).

### Resources

**Figure 2:**
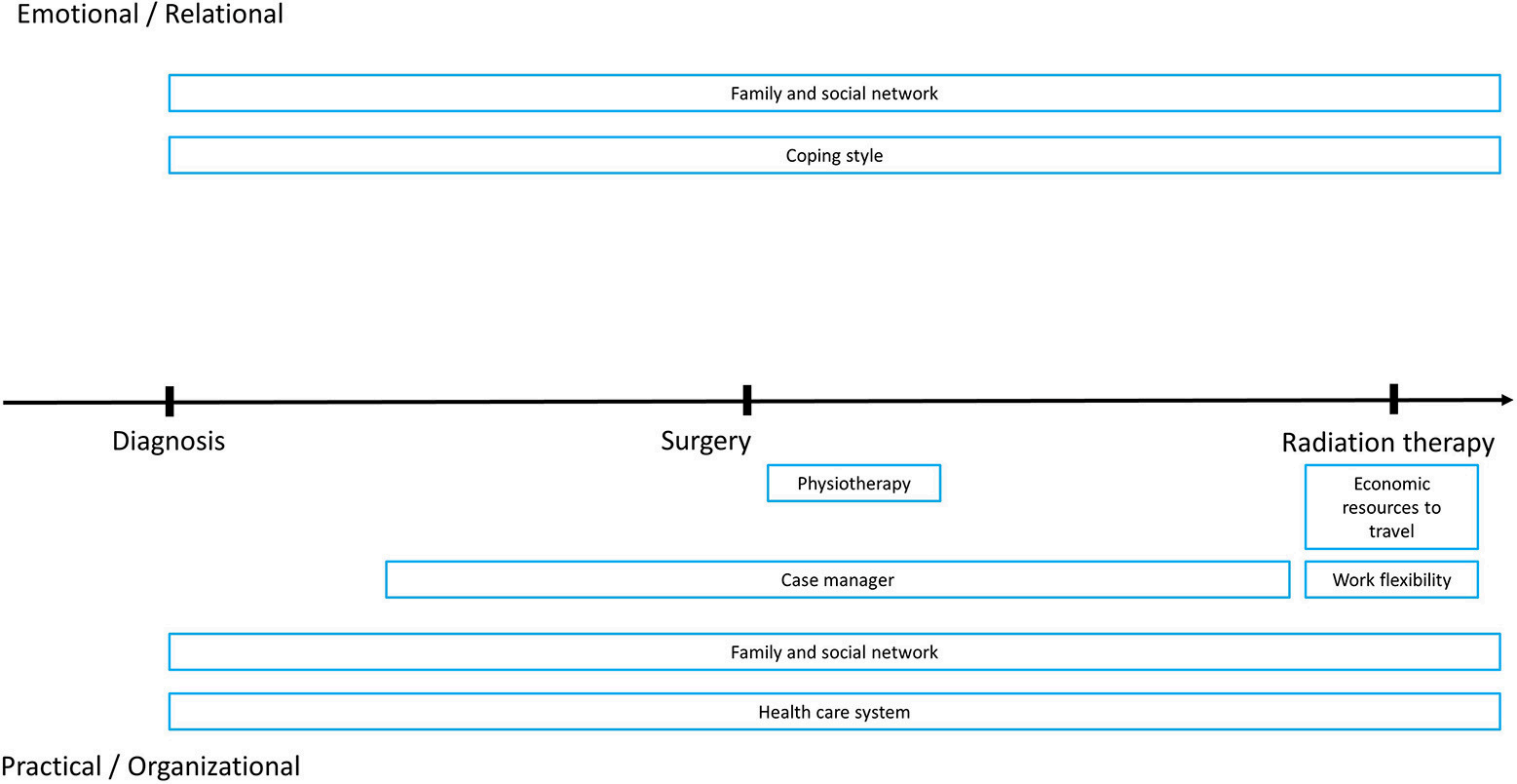
Graphic representation of reported resources by temporal dimensions (diagnosis, surgery, radiation therapy) and type (Emotional/relational, Practical/organizational).

Resources enounced were grouped into themes based on patients' narratives. To facilitate interpretation of data, they were displayed along two dimensions: one ranging from practical/organizational to emotional/relational, while the other ranging from diagnosis to surgery to radiation therapy (Figure 2).

Family and social support network (7 participants): The presence of supportive partners, relatives, and friends was perceived as a major resource in all phases from diagnosis to therapies. They provide support both in the emotional/relational context (4 participants; empathic listening, care and support, help in deciding) as well as in the practical/organizational (5 participants; help in the choice of the specialist, of the hospital or of the therapy, support in collecting relevant information, help with self-management). Friendly relations with fellow patients in the waiting room of radiation therapy were also cited. For instance, Patient 6 reported: “*My relatives' network was of help to get access to some clinical structures. […]. They gave me psychological support*.” Similarly, Patient 4 says: “*My social network was important for the contact with structures*.” Patient 7 said: “*It was good to have a social network to ask information on where to go. […]. I feel sympathetic for those who are ‘on the same boat.’ You can share. […] My wife and my friends are supportive*.” Patient 2 stated: “*I had my daughter helping at home to manage the catheter. […] My family and my friends helped me to choose and gather information, e.g., they came with me for consultations*.” Patient 4 said: “*I can communicate and share with my family and friends. We talk on the phone everyday*” (Relational; all phases).

Coping style (4 participants): individual coping styles (fighting spirit, optimism, being patient and determined, and religiosity) were cited as a resource, in particular during therapy. For instance, Patient 9 said: “*I showed courage, won the fear of the unknown. I had never been admitted to an hospital before this surgery*.” Patient 6 said: “*I have strength and optimism*.” (Emotional/relational; all phases).

Economic resources to travel (2 participants): the economic possibility to travel during radiation therapy or to live near the hospital after surgery and during radiation therapy were perceived as resources that could decrease distress (Practical/organizational; after surgery, during radiation therapy).

Health-care system (1 participant): free medical assistance through the healthcare system (Practical/organizational; all phases).

Work management (3 participants): possibility to have flexible working schedules or working environments, and the chance to ask for disease leave (Practical/organizational; during radiation therapy).

Physiotherapists (2 participants): the availability of these healthcare professionals in the staff led to greater self-confidence in managing dysuria and prompt rehabilitation of urinary functions (Practical/organizational; after surgery).

Case-manager (1 participant): the presence of a case-manager was perceived as a reference to organize appointments and consultations in the hospital. Patient 5 described: “*she was assertive, maternal, reassuring, someone that tells what you the do's and dont's*” (Practical/organizational; all phases).

## Discussion

The present study was designed to assess aspects of empowerment in prostate cancer individuals in order to develop a web-based eHealth platform to improve self-management abilities and to promote cancer patients' empowerment. Patients' communication and decision-making models implemented with their clinicians as well as difficulties, needs and resources were explored. A qualitative methodology was adopted to elicit patients' individual experiences, allowing to unveil patients' points of view. Semi-structured interviews were run with 10 patients and then a thematic analysis was performed.

With respect to the quality of communication, overall patients participating in the present study experienced different communicative interactions with their clinicians. Directive communication styles were associated with a passive role in communication, and in part of the decision-making process. In two cases, experiencing directive communication was considered so unsatisfactory that it represented a reason for the patient to change the treating physician. In those patients who need to collect and receive information to engage in the decision-making process, the directive/informative style of communication appeared as a signal of detachment from clinicians, undermining the therapeutic alliance. In six cases, this resulted in changes of specialist before deciding for treatment (in four cases more than two specialists were consulted), thus delaying therapy and increasing stress for the patient. However, given that patients were recruited in a comprehensive cancer center, it is possible that there is a sampling bias since unsatisfied patients may be more likely to converge to renowned centers or bigger hospitals.

Critically, shared decision-making is reflected only in three of the narratives of the present sample thus suggesting that the model of caring is still perceived as either placing the responsibility on the physician or as placing it on the patient and his family.

Consistently, a great part of the unmet needs that were reported by the present sample were related to communication and decision-making issues (e.g., Choice of specialist, Clinicians' support in self-management, Surgical consequences and side-effects, Preparation for radiation therapy). Patients felt they often lacked sufficient information in order to make decisions e.g., on the therapeutic approach which suited them better or in order to take decisions related to self-management during or after therapy.

Results indicate that a current relevant issue for the population involved in this study concerns the relation with healthcare professionals. In prostate cancer, the patient-physician relation represents an important factor for decision-making, as highlighted also in quantitative studies (Cegala et al., 2008; Orom et al., 2014). In the literature, the patients' lack of sufficient information and the failure to establish a trustful relation with clinicians was associated with precluding participation in decision-making (Davison et al., 1995; Davison and Degner, 1997; Cutica et al., 2014).

Henry et al. (2015) reported that physicians often devote little time to discuss cancer diagnosis, and quickly skip to describe treatment options. In a Japanese study (Ishikawa et al., 2002) on patients-physicians interactions, patients reported that physician's direction and encouragement were negatively associated with patient satisfaction. However, patients who asked more questions were less satisfied with the consultation, indicating that information may not be sufficient to establish a trustful relation with physicians. These data point to the importance of supporting patient empowerment and patient-clinicians relations from the very beginning of the careflow. An Australian-Canadian qualitative study found that compassion, reassurance and humor were pivotal factors to develop a feeling of trust in their physician (Oliffe and Thorne, 2007). Furthermore, the use of open-ended questions (Ishikawa et al., 2002) and explaining the decision-making process before treatment options may promote a patient-centered communication (Henry et al., 2015).

Patients from the present sample stressed the importance of taking into account their view and their preferences when discussing the choice of therapies with healthcare providers, as well as the need to be prepared for possible complications or side effects. This suggests that there is room for improvements in delivering the appropriate information for each step throughout the careflow. Patient-centered care is now widely recognized benchmarks of quality care for chronic cancer patients (Epstein and Street, 2007; McCorkle et al., 2011) associated with greater quality of life and adjustment to disease. In the case of prostate cancer, the patient's need to be involved in the care path definition (Davison and Degner, 1997) is extremely important also because of the important side effects that patients have to face in the daily management of the disease.

Clinical and eHealth interventions to support patient empowerment may provide feedback to clinicians in order to tailor communication according to the patient's characteristics (Kondylakis et al., 2012, 2013, 2014; Wilkes et al., 2013; Gorini et al., 2015). Furthermore, initial information on cancer disease may be offered through eHealth platforms, helping the patient to formulate and note relevant questions prior to medical consultation (Kondylakis et al., 2014, 2015). A possible implementation of these suggestions in a comprehensive cancer platform for patient empowerment is provided by Kazantzaki et al. (2016) and by Renzi et al. (2016).

Clinical consultation material or eHealth platforms may include tools that help patients and clinicians visualize the careflow and the decision-making process. Concerning information provision and decision-making support, decision aid tools may be provided to patients' and clinicians to support decision-making and to promote a decision-making style respectful of the patient's preferences (Charles et al., 1999).

Another relevant finding of the study indicated that lack of knowledge and lack of reference figures seem to have an important role in determining relational difficulties with healthcare providers and decreasing patient empowerment, while this should be a primary goal to prevent low adherence to treatments, avoidable side effects, and unmet patients' needs. When information is perceived as available or when a reference figure is identified in the clinical staff (e.g., case-manager), patients perceived to be supported. eHealth platforms may consider supporting the role of the case-manager, providing useful tools for planning and scheduling appointments in the careflow, monitoring (e.g., how to monitor drainage), reference contacts for different issues that may arise (e.g., post-surgical complications vs. changing a scheduled clinical consultation).

Involving the patients in the definition of treatment implies to take into consideration important aspects of their daily life, which can play a crucial role in therapeutic alliance. Practical aspects related to daily life management possibilities, individual and social resources, should be discussed in the clinical consultation, and should be integrated in the design of eHealth platforms. Travelling to receive therapies or maintaining the job position are important factors that need to be considered in the decision-making process. Patients that are far from home could need emotional and psychological support if they face the care treatment alone, especially when they undergo surgery and experience post-surgical pain or complications. Also in this case, eHealth applications may serve as a tool to support the alliance with the clinicians (Berry et al., 2011; Cleeland et al., 2011; Cook et al., 2013; Vonk Noordegraaf et al., 2014).

In line with the literature on prostate cancer (Mehnert et al., 2010; Queenan et al., 2010; Zhou et al., 2010; Paterson et al., 2013), our study pointed out the perceived importance of individual factors (e.g., coping) as well as the perceived availability of a familiar and social network to decrease distress in prostate cancer patients throughout the careflow. Importantly, coping style seems to mediate long-term effects of social support (Zhou et al., 2010). This highlights the importance of monitoring both individual resources, as well as social support from the beginning of the careflow, with the perspective of a dynamic and flexible process (Ambrosio et al., 2015) where conditions that may undermine patient's well-being are identified and appropriate support is offered.

The present study explored different aspects of cancer patient empowerment and investigated their perceived needs and resources. Indications for clinical practice and for the development of eHealth platforms were provided. Importantly, further studies should evaluate the relevance of implementing these indications in the promotion of patient empowerment, self-management abilities and health-related quality of life.

Patient-centered care is now widely recognized benchmarks of quality care for chronic cancer patients (Epstein and Street, 2007; McCorkle et al., 2011) associated with greater quality of life and adjustment to disease. In the case of prostate cancer, the patient's need to be involved in the care path definition (Davison and Degner, 1997) is extremely important also because of the important side effects that patients have to face in the daily management of the disease.

A limitation of the present study is that patients were recruited in a single comprehensive cancer center in northern Italy. Therefore, the sample may not be representative of the prostate cancer population in smaller or other regional centers, both in terms of information provision and decision-making processes as well as in terms of needs and resources. Future studies may address whether and to what extent there is a difference between patients followed in cancer centers and those treated in local hospital. Furthermore, in order to explore the living experience of prostate cancer patients undergoing radiation therapy treatment, a qualitative methodology was used. This provided an in-depth perspective on different aspects of patient empowerment, however a qualitative approach and the use of small samples does not allow to capture those factors which may be significantly associated with the empowerment of prostate cancer patients. Further research may address the issues emerged from the present work using a quantitative methodology.

## Author contributions

CR, CF, SO, KM, DZ, GP, and BJ: Designed the study; CR, SO, and CF: Acquired the data; CR, CF, SO, OA, and DR: Analyzed the data; CR, CF, DZ, OA, DR, BJ, and GP: Discussed the data. All authors participated in the drafting of the manuscript and approved its final version.

### Conflict of interest statement

The authors declare that the research was conducted in the absence of any commercial or financial relationships that could be construed as a potential conflict of interest.
